# Supplementary material for: New Insights into Plant TPK Ion Channel Evolution
Source: Plants (Basel). 2021 Oct 28;10(11):2328. doi: 10.3390/plants10112328 (PMC8619664; doi:10.3390/plants10112328)
Supplement: Supplementary file 1 [file plants-1414539-supplementary-Figures S1-S12.pdf › plants-1414539-supplementary/plants-1414539-supplementary-Figures S1-S12.pdf]

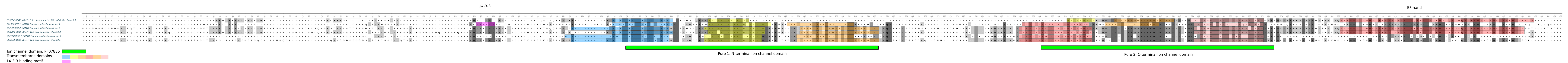

Figure S1. Arabidopsis thaliana TPK proteins alignment. EF-hand and 14-3-3 domains, transmembrane regions and pores are highlighted.

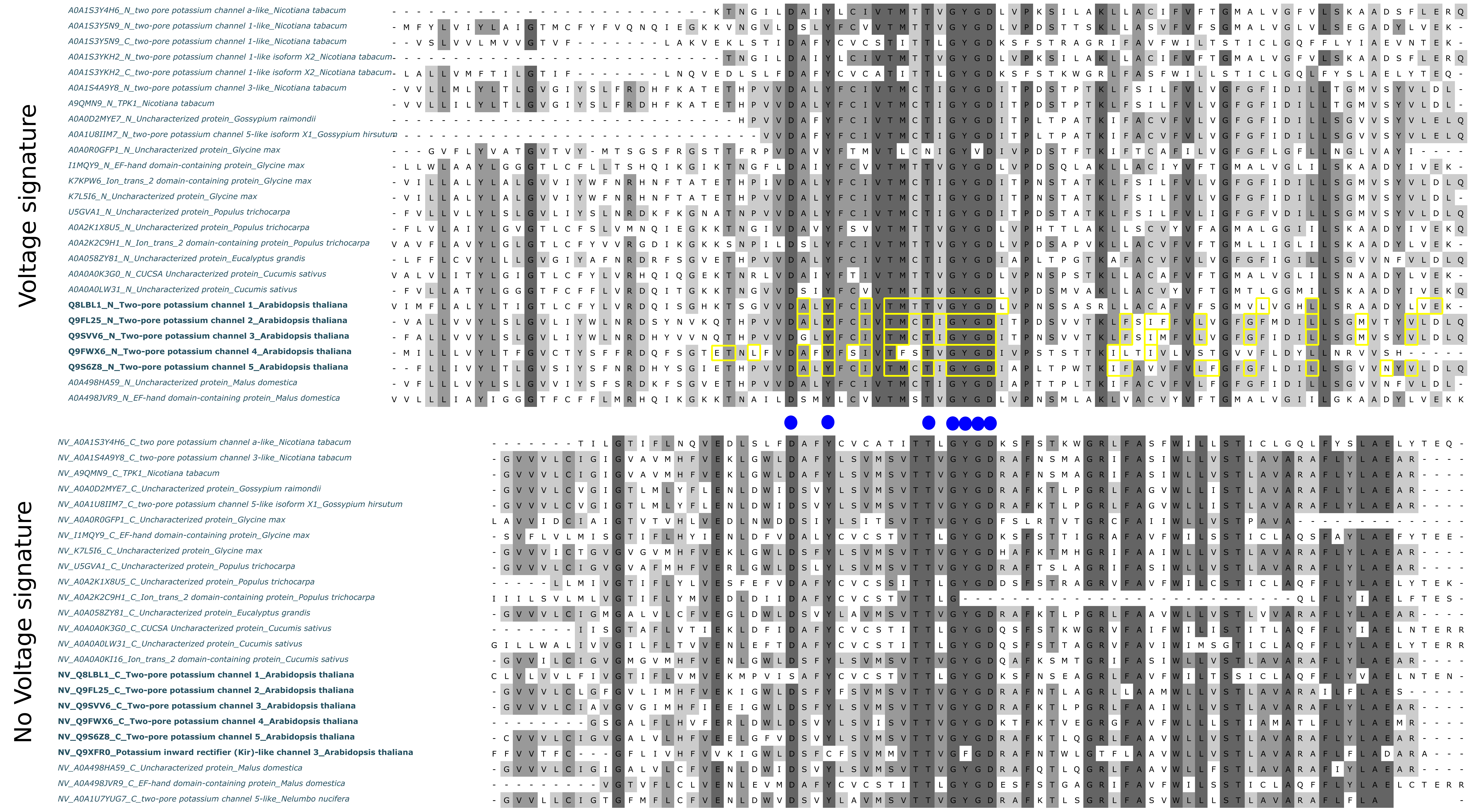

Figure S2. Alignment of the 1st (with VDC signature) and 2nd pore (without VDC signature) from the dicotyledon plants TPK homologues. Model species Arabidopsis thaliana proteins are highlighted in bold. VDC signature – in yellow; conserved residues are marked with blue dots.

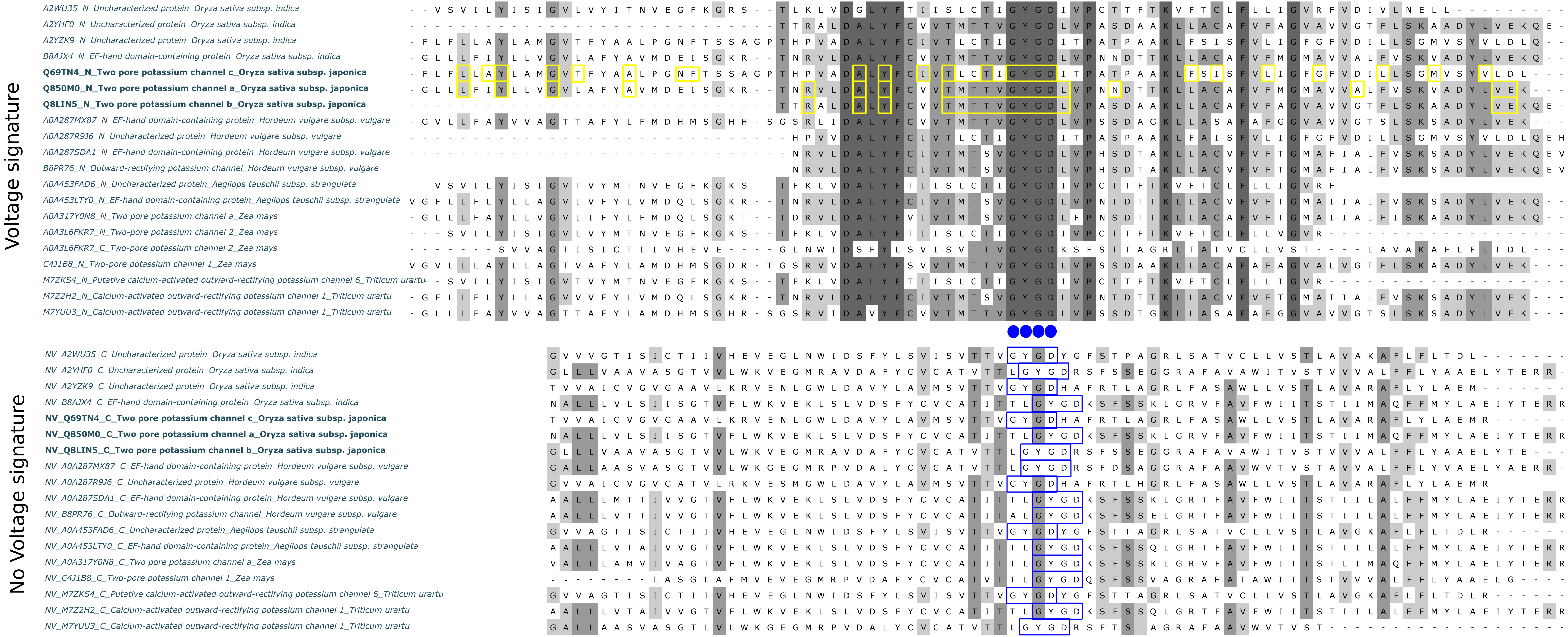

Figure S3. Alignment of the 1st (with VDC signature) and 2nd pore (without VDC signature) from the monocotyledon plants TPK homologues. Model species Oryza sativa subsp. japonica proteins are highlighted in bold. GYGD selectivity filter motif is highlighted in blue; VDC signature – in yellow; conserved residues are marked with blue dots.

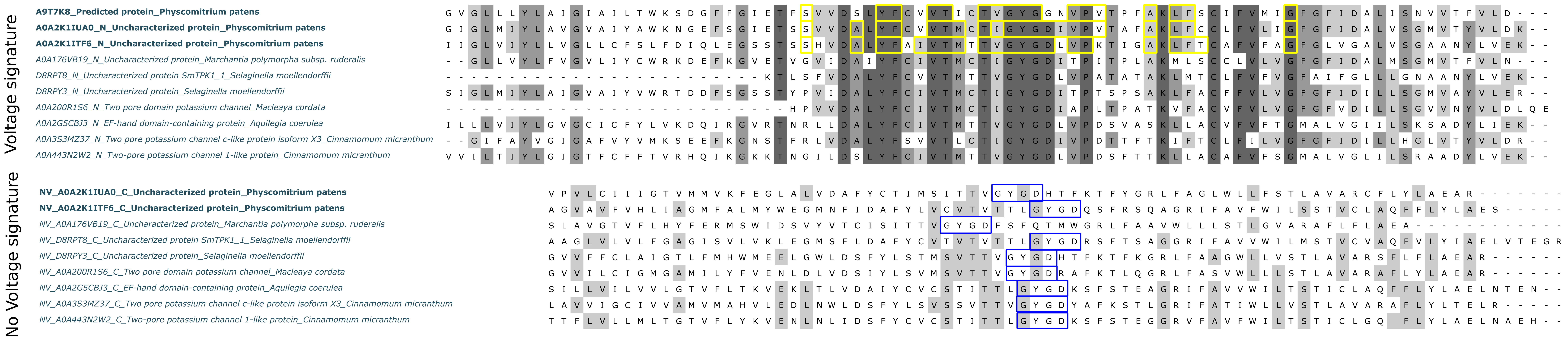

Figure S4. Alignment of the 1st (with VDC signature) and 2nd pore (without VDC signature) from the first land conquers ("low" plants) TPK homologues. GYGD selectivity filter motif is highlighted in blue; VDC signature – in yellow; Model species Physcomitrella patens proteins are highlighted in bold.

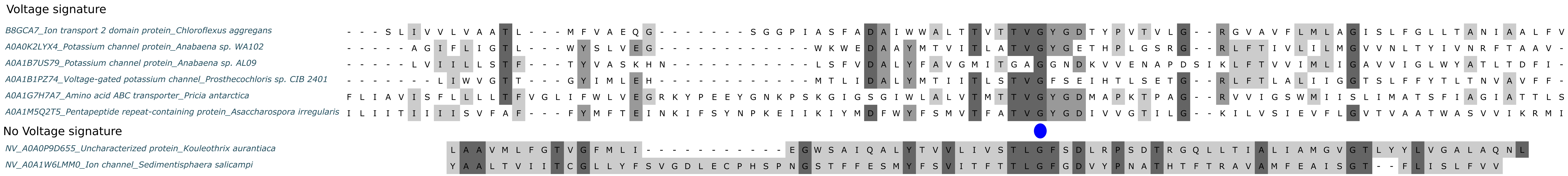

Figure S5. Alignment of the 1st (with VDC signature) and 2nd pore (without VDC signature) from the photosynthetic bacteria TPK homologues. While some species do have a complete GYGD motif, only one residue is conserved (marked with a blue dot).

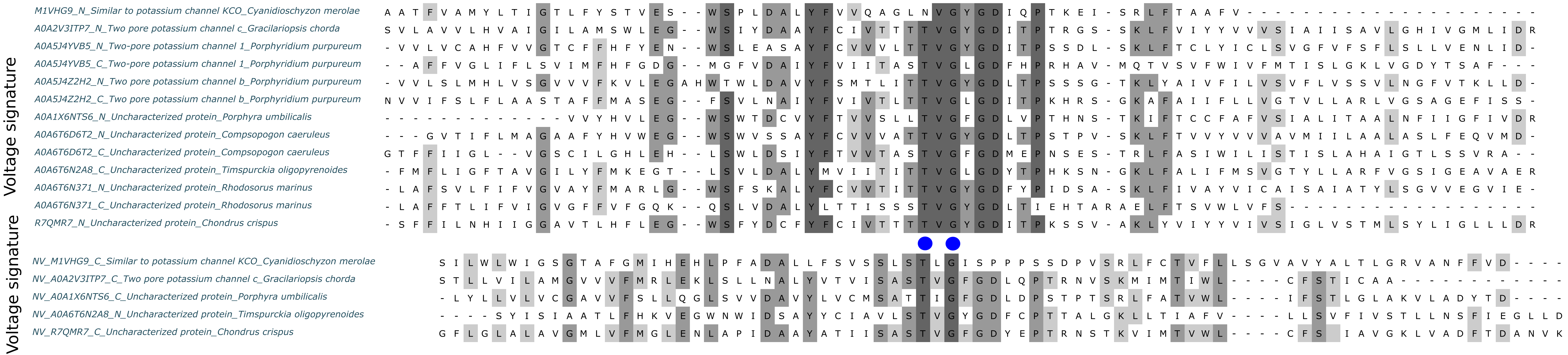

Figure S6. Alignment of the 1st (with VDC signature) and 2nd pore (without VDC signature) from the red algae TPK homologues. The majority of species do have a complete GYGD motif, however, only some residues are conserved between two pores (marked with a blue dot). The “Y” residue in the GYGD selectivity filter motif is often replaced with L or F.

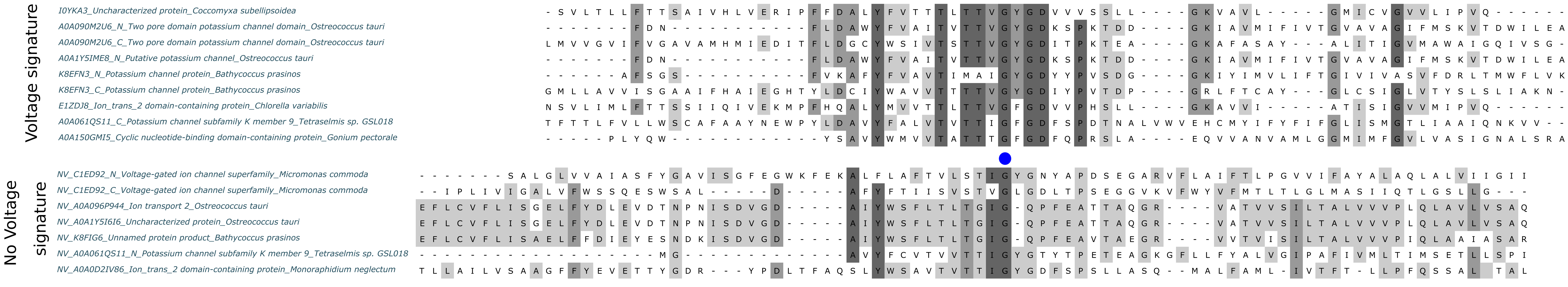

Figure S7. Alignment of the 1st (with VDC signature) and 2nd pore (without VDC signature) from the green algae TPK homologues. The majority of species do have a complete GYGD motif, however, only one residue is conserved between two pores (marked with a blue dot). The “Y” residue in the GYGD selectivity filter motif is often replaced with F (the 1st pore) or L (the 2nd pore).

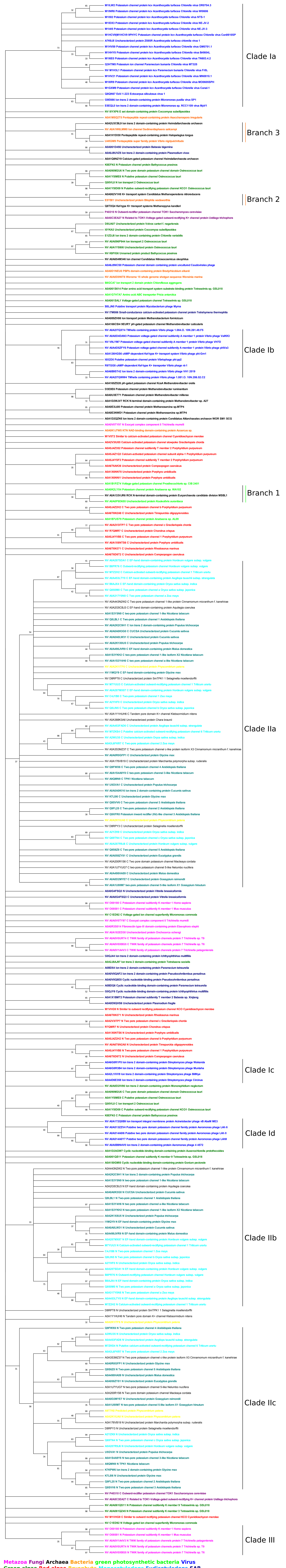

S_Figure 8. Phylogeny estimation of the ion channel domains from different taxa. 1st pore (N-terminal ion channel domain, N) and 2nd pore (C-terminal ion channel domain, C) sequences were extracted TPK proteins of selected taxa have been used for phylogeny reconstruction with Neighbor-Joining method and JTT substitution model. Absence of the voltage-dependent channels (VDC) signature is depicted as "NV_" before the protein's ID.

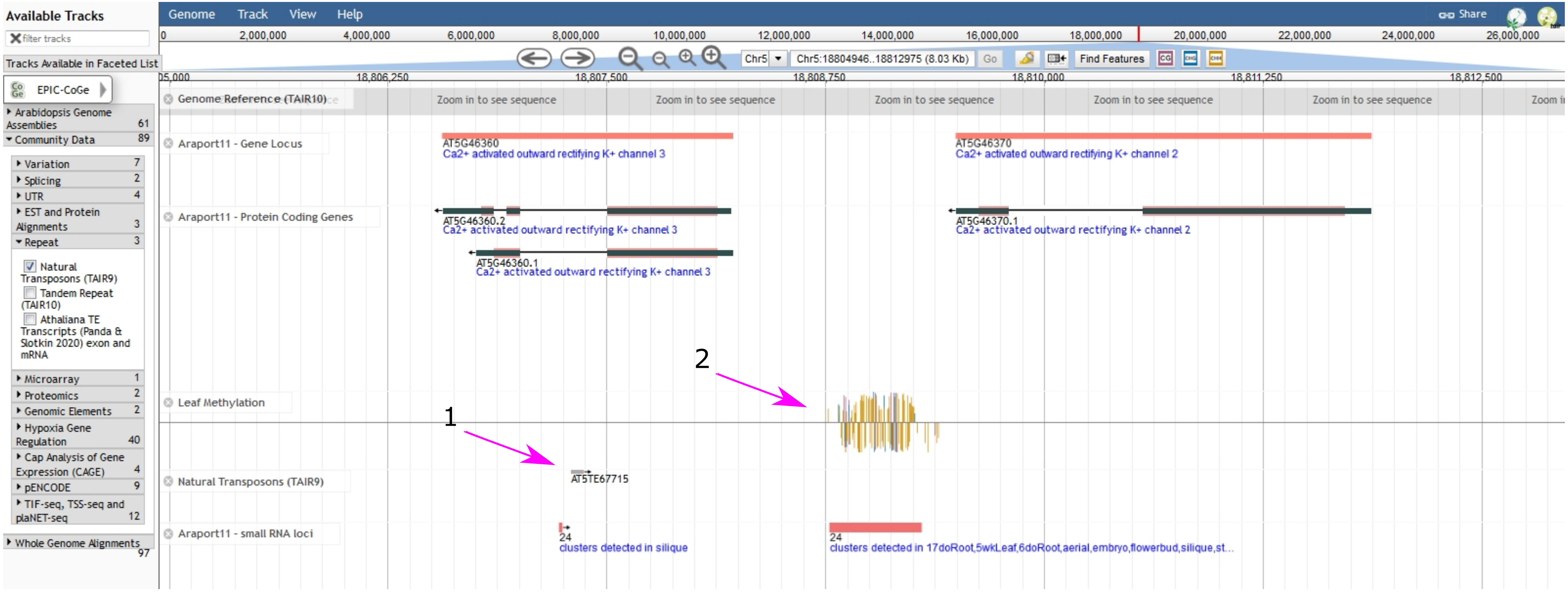

Figure S9. Genome browser look on Arabidopsis KCO3 gene. 1 – transposable element (TE) ID AT5TE67715; 2 – methylation of the KCO3 promoter.

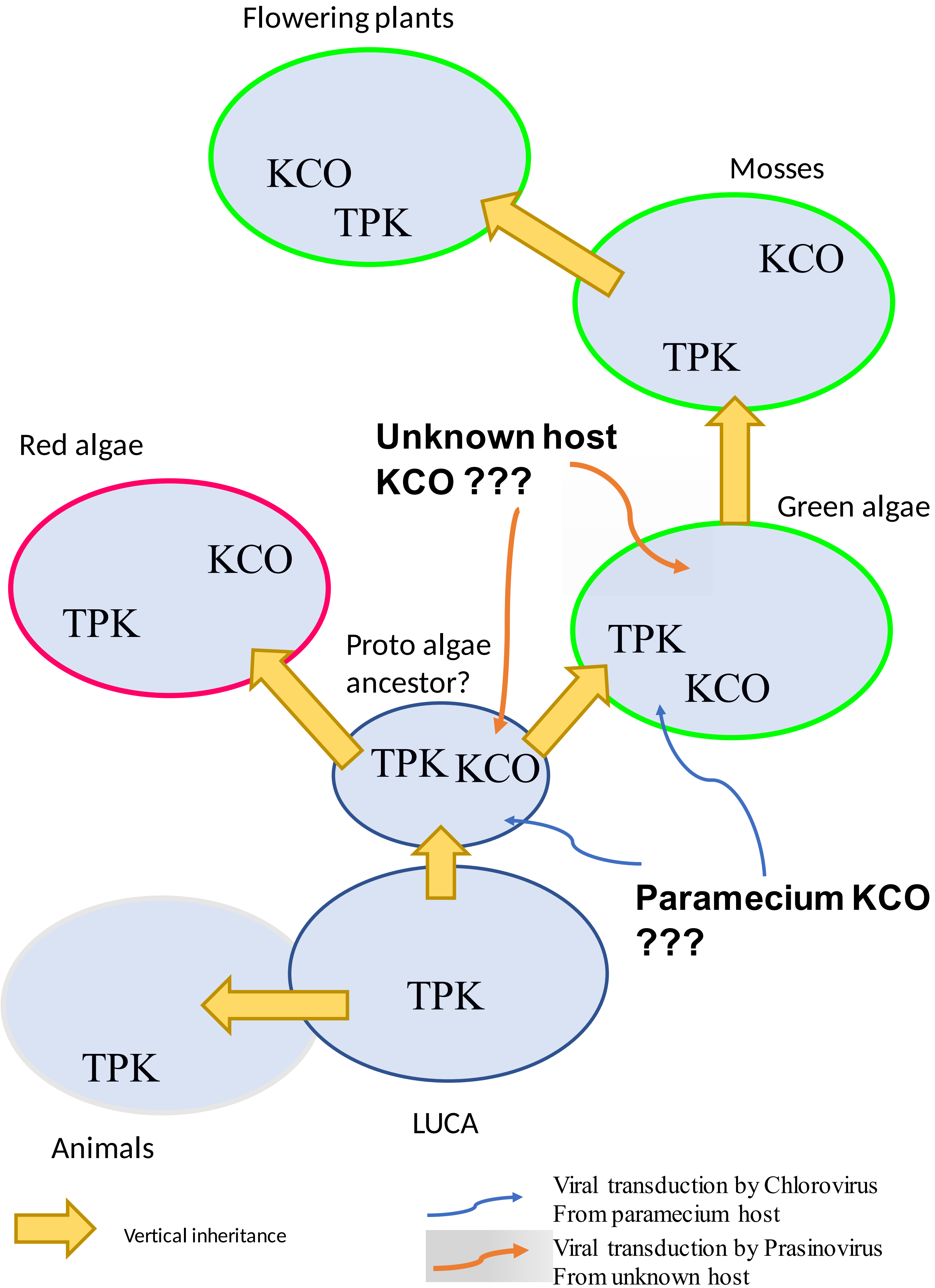

Figure S10. Proposed model of the TPK channels evolution

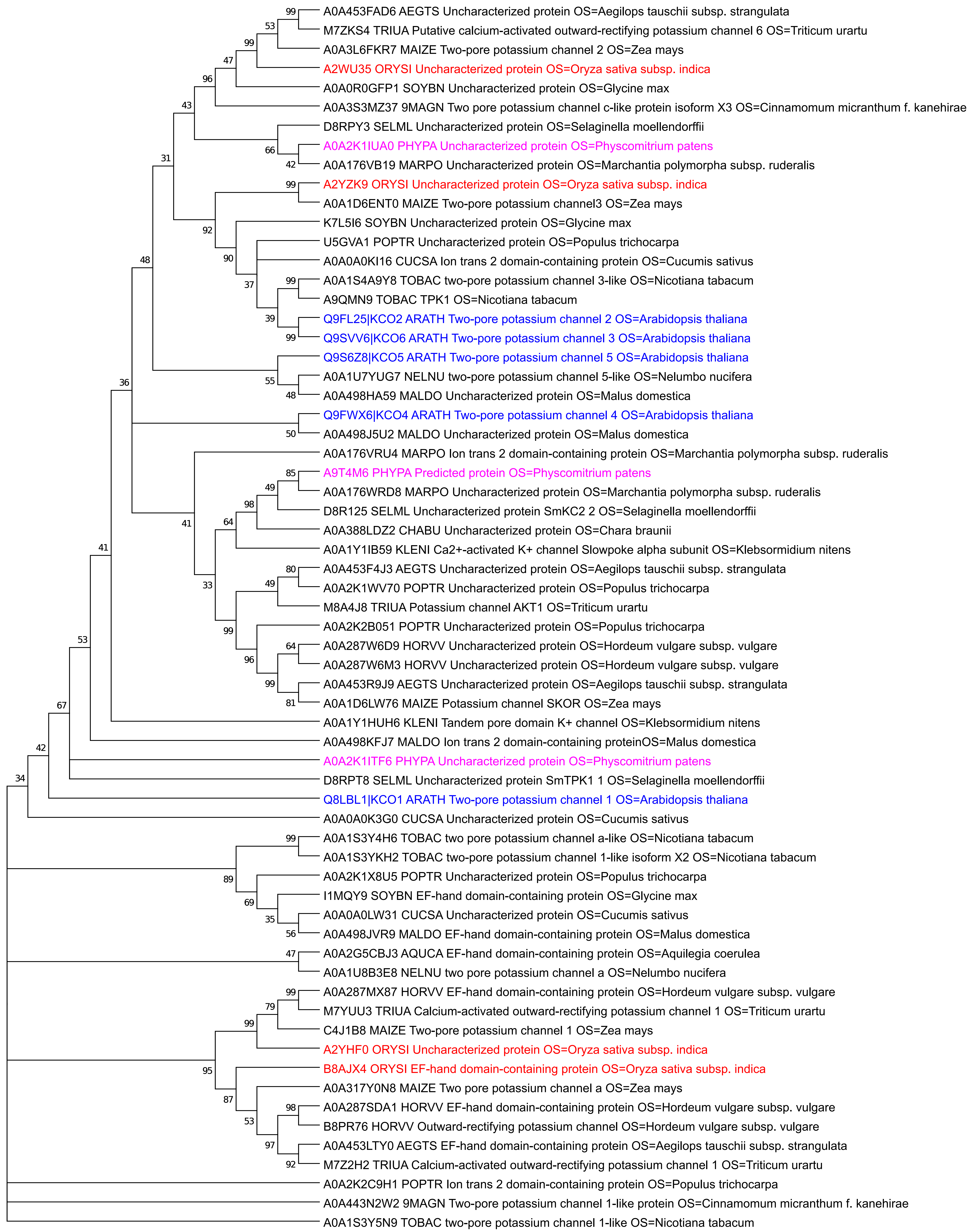
A0A453FAD6 AEGTS Uncharacterized protein OS=Aegilops tauschii subsp. strangulata
M7ZKS4 TRIUA Putative calcium-activated outward-rectifying potassium channel 6 OS=Triticum urartu
A0A3L6FKR7 MAIZE Two-pore potassium channel 2 OS=Zea mays
A2WU35 ORYSI Uncharacterized protein OS=Oryza sativa subsp. indica
A0A0R0GFP1 SOYBN Uncharacterized protein OS=Glycine max
A0A3S3MZ37 9MAGN Two pore potassium channel c-like protein isoform X3 OS=Cinnamomum micranthum f. kanehirae
D8RPY3 SELML Uncharacterized protein OS=Selaginella moellendorffii
A0A2K1IUA0 PHYPA Uncharacterized protein OS=Physcomitrium patens
A0A176VB19 MARPO Uncharacterized protein OS=Marchantia polymorpha subsp. ruderalis
A2YZK9 ORYSI Uncharacterized protein OS=Oryza sativa subsp. indica
A0A1D6ENT0 MAIZE Two-pore potassium channel3 OS=Zea mays
K7L5I6 SOYBN Uncharacterized protein OS=Glycine max
U5GVA1 POPTR Uncharacterized protein OS=Populus trichocarpa
A0A0A0KI16 CUCSA Ion trans 2 domain-containing protein OS=Cucumis sativus
A0A1S4A9Y8 TOBAC two-pore potassium channel 3-like OS=Nicotiana tabacum
A9QMN9 TOBAC TPK1 OS=Nicotiana tabacum
Q9FL25|KCO2 ARATH Two-pore potassium channel 2 OS=Arabidopsis thaliana
Q9SVV6|KCO6 ARATH Two-pore potassium channel 3 OS=Arabidopsis thaliana
Q9S6Z8|KCO5 ARATH Two-pore potassium channel 5 OS=Arabidopsis thaliana
A0A1U7YUG7 NELNU two-pore potassium channel 5-like OS=Nelumbo nucifera
A0A498HA59 MALDO Uncharacterized protein OS=Malus domestica
Q9FWX6|KCO4 ARATH Two-pore potassium channel 4 OS=Arabidopsis thaliana
A0A498J5U2 MALDO Uncharacterized protein OS=Malus domestica
A0A176VRU4 MARPO Ion trans 2 domain-containing protein OS=Marchantia polymorpha subsp. ruderalis
A9T4M6 PHYPA Predicted protein OS=Physcomitrium patens
A0A176WRD8 MARPO Uncharacterized protein OS=Marchantia polymorpha subsp. ruderalis
D8R125 SELML Uncharacterized protein SmKC2 2 OS=Selaginella moellendorffii
A0A388LDZ2 CHABU Uncharacterized protein OS=Chara braunii
A0A1Y1IB59 KLENI Ca2+-activated K+ channel Slowpoke alpha subunit OS=Klebsormidium nitens
A0A453F4J3 AEGTS Uncharacterized protein OS=Aegilops tauschii subsp. strangulata
A0A2K1WV70 POPTR Uncharacterized protein OS=Populus trichocarpa
M8A4J8 TRIUA Potassium channel AKT1 OS=Triticum urartu
A0A2K2B051 POPTR Uncharacterized protein OS=Populus trichocarpa
A0A287W6D9 HORVV Uncharacterized protein OS=Hordeum vulgare subsp. vulgare
A0A287W6M3 HORVV Uncharacterized protein OS=Hordeum vulgare subsp. vulgare
A0A453R9J9 AEGTS Uncharacterized protein OS=Aegilops tauschii subsp. strangulata
A0A1D6LW76 MAIZE Potassium channel SKOR OS=Zea mays
A0A1Y1HUH6 KLENI Tandem pore domain K+ channel OS=Klebsormidium nitens
A0A498KFJ7 MALDO Ion trans 2 domain-containing proteinOS=Malus domestica
A0A2K1ITF6 PHYPA Uncharacterized protein OS=Physcomitrium patens
D8RPT8 SELML Uncharacterized protein SmTPK1 1 OS=Selaginella moellendorffii
Q8LBL1|KCO1 ARATH Two-pore potassium channel 1 OS=Arabidopsis thaliana
A0A0A0K3G0 CUCSA Uncharacterized protein OS=Cucumis sativus
A0A1S3Y4H6 TOBAC two pore potassium channel a-like OS=Nicotiana tabacum
A0A1S3YKH2 TOBAC two-pore potassium channel 1-like isoform X2 OS=Nicotiana tabacum
A0A2K1X8U5 POPTR Uncharacterized protein OS=Populus trichocarpa
I1MQY9 SOYBN EF-hand domain-containing protein OS=Glycine max
A0A0A0LW31 CUCSA Uncharacterized protein OS=Cucumis sativus
A0A498JVR9 MALDO EF-hand domain-containing protein OS=Malus domestica
A0A2G5CBJ3 AQUCA EF-hand domain-containing protein OS=Aquilegia coerulea
A0A1U8B3E8 NELNU two pore potassium channel a OS=Nelumbo nucifera
A0A287MX87 HORVV EF-hand domain-containing protein OS=Hordeum vulgare subsp. vulgare
M7YUU3 TRIUA Calcium-activated outward-rectifying potassium channel 1 OS=Triticum urartu
C4J1B8 MAIZE Two-pore potassium channel 1 OS=Zea mays
A2YHF0 ORYSI Uncharacterized protein OS=Oryza sativa subsp. indica
B8AJX4 ORYSI EF-hand domain-containing protein OS=Oryza sativa subsp. indica
A0A317Y0N8 MAIZE Two pore potassium channel a OS=Zea mays
A0A287SDA1 HORVV EF-hand domain-containing protein OS=Hordeum vulgare subsp. vulgare
B8PR76 HORVV Outward-rectifying potassium channel OS=Hordeum vulgare subsp. vulgare
A0A453LTY0 AEGTS EF-hand domain-containing protein OS=Aegilops tauschii subsp. strangulata
M7Z2H2 TRIUA Calcium-activated outward-rectifying potassium channel 1 OS=Triticum urartu
A0A2K2C9H1 POPTR Ion trans 2 domain-containing protein OS=Populus trichocarpa
A0A443N2W2 9MAGN Two-pore potassium channel 1-like protein OS=Cinnamomum micranthum f. kanehirae
A0A1S3Y5N9 TOBAC two-pore potassium channel 1-like OS=Nicotiana tabacum

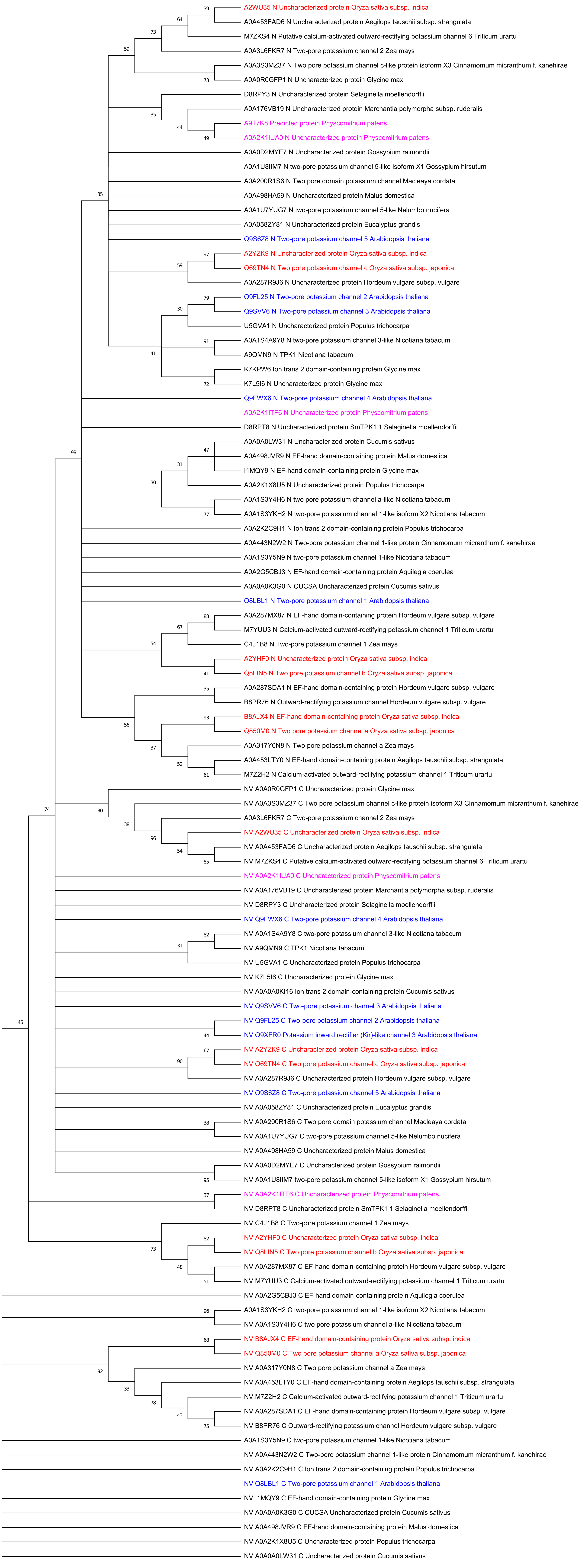

A2WU35 N Uncharacterized protein Oryza sativa subsp. indica
A0A453FAD6 N Uncharacterized protein Aegilops tauschii subsp. strangulata
M7ZKS4 N Putative calcium-activated outward-rectifying potassium channel 6 Triticum urartu
A0A3L6FKR7 N Two-pore potassium channel 2 Zea mays
A0A3S3MZ37 N Two pore potassium channel c-like protein isoform X3 Cinnamomum micranthum f. kanehirae
A0A0R0GFP1 N Uncharacterized protein Glycine max
D8RPY3 N Uncharacterized protein Selaginella moellendorffii
A0A176VB19 N Uncharacterized protein Marchantia polymorpha subsp. ruderalis
A9T7K8 Predicted protein Physcomitrium patens
A0A2K1IUA0 N Uncharacterized protein Physcomitrium patens
A0A0D2MYE7 N Uncharacterized protein Gossypium raimondii
A0A1U8IIM7 N two-pore potassium channel 5-like isoform X1 Gossypium hirsutum
A0A200R1S6 N Two pore domain potassium channel Macleaya cordata
A0A498HA59 N Uncharacterized protein Malus domestica
A0A1U7YUG7 N two-pore potassium channel 5-like Nelumbo nucifera
A0A058ZY81 N Uncharacterized protein Eucalyptus grandis
Q9S6Z8 N Two-pore potassium channel 5 Arabidopsis thaliana
A2YZK9 N Uncharacterized protein Oryza sativa subsp. indica
Q69TN4 N Two pore potassium channel c Oryza sativa subsp. japonica
A0A287R9J6 N Uncharacterized protein Hordeum vulgare subsp. vulgare
Q9FL25 N Two-pore potassium channel 2 Arabidopsis thaliana
Q9SVV6 N Two-pore potassium channel 3 Arabidopsis thaliana
U5GVA1 N Uncharacterized protein Populus trichocarpa
A0A1S4A9Y8 N two-pore potassium channel 3-like Nicotiana tabacum
A9QMN9 N TPK1 Nicotiana tabacum
K7KPW6 Ion trans 2 domain-containing protein Glycine max
K7L5I6 N Uncharacterized protein Glycine max
Q9FWX6 N Two-pore potassium channel 4 Arabidopsis thaliana
A0A2K1ITF6 N Uncharacterized protein Physcomitrium patens
D8RPT8 N Uncharacterized protein SmTPK1 1 Selaginella moellendorffii
A0A0A0LW31 N Uncharacterized protein Cucumis sativus
A0A498JVR9 N EF-hand domain-containing protein Malus domestica
I1MQY9 N EF-hand domain-containing protein Glycine max
A0A2K1X8U5 N Uncharacterized protein Populus trichocarpa
A0A1S3Y4H6 N two pore potassium channel a-like Nicotiana tabacum
A0A1S3YKH2 N two-pore potassium channel 1-like isoform X2 Nicotiana tabacum
A0A2K2C9H1 N Ion trans 2 domain-containing protein Populus trichocarpa
A0A443N2W2 N Two-pore potassium channel 1-like protein Cinnamomum micranthum f. kanehirae
A0A1S3Y5N9 N two-pore potassium channel 1-like Nicotiana tabacum
A0A2G5CBJ3 N EF-hand domain-containing protein Aquilegia coerulea
A0A0A0K3G0 N CUCSA Uncharacterized protein Cucumis sativus
Q8LBL1 N Two-pore potassium channel 1 Arabidopsis thaliana
A0A287MX87 N EF-hand domain-containing protein Hordeum vulgare subsp. vulgare
M7YUU3 N Calcium-activated outward-rectifying potassium channel 1 Triticum urartu
C4J1B8 N Two-pore potassium channel 1 Zea mays
A2YHF0 N Uncharacterized protein Oryza sativa subsp. indica
Q8LIN5 N Two pore potassium channel b Oryza sativa subsp. japonica
A0A287SDA1 N EF-hand domain-containing protein Hordeum vulgare subsp. vulgare
B8PR76 N Outward-rectifying potassium channel Hordeum vulgare subsp. vulgare
B8AJX4 N EF-hand domain-containing protein Oryza sativa subsp. indica
Q850M0 N Two pore potassium channel a Oryza sativa subsp. japonica
A0A317Y0N8 N Two pore potassium channel a Zea mays
A0A453LTY0 N EF-hand domain-containing protein Aegilops tauschii subsp. strangulata
M7Z2H2 N Calcium-activated outward-rectifying potassium channel 1 Triticum urartu
NV A0A0R0GFP1 C Uncharacterized protein Glycine max
NV A0A3S3MZ37 C Two pore potassium channel c-like protein isoform X3 Cinnamomum micranthum f. kanehirae
A0A3L6FKR7 C Two-pore potassium channel 2 Zea mays
NV A2WU35 C Uncharacterized protein Oryza sativa subsp. indica
NV A0A453FAD6 C Uncharacterized protein Aegilops tauschii subsp. strangulata
NV M7ZKS4 C Putative calcium-activated outward-rectifying potassium channel 6 Triticum urartu
NV A0A2K1IUA0 C Uncharacterized protein Physcomitrium patens
NV A0A176VB19 C Uncharacterized protein Marchantia polymorpha subsp. ruderalis
NV D8RPY3 C Uncharacterized protein Selaginella moellendorffii
NV Q9FWX6 C Two-pore potassium channel 4 Arabidopsis thaliana
NV A0A1S4A9Y8 C two-pore potassium channel 3-like Nicotiana tabacum
NV A9QMN9 C TPK1 Nicotiana tabacum
NV U5GVA1 C Uncharacterized protein Populus trichocarpa
NV K7L5I6 C Uncharacterized protein Glycine max
NV A0A0A0KI16 Ion trans 2 domain-containing protein Cucumis sativus
NV Q9SVV6 C Two-pore potassium channel 3 Arabidopsis thaliana
NV Q9FL25 C Two-pore potassium channel 2 Arabidopsis thaliana
NV Q9XFR0 Potassium inward rectifier (Kir)-like channel 3 Arabidopsis thaliana
NV A2YZK9 C Uncharacterized protein Oryza sativa subsp. indica
NV Q69TN4 C Two pore potassium channel c Oryza sativa subsp. japonica
NV A0A287R9J6 C Uncharacterized protein Hordeum vulgare subsp. vulgare
NV Q9S6Z8 C Two-pore potassium channel 5 Arabidopsis thaliana
NV A0A058ZY81 C Uncharacterized protein Eucalyptus grandis
NV A0A200R1S6 C Two pore domain potassium channel Macleaya cordata
NV A0A1U7YUG7 C two-pore potassium channel 5-like Nelumbo nucifera
NV A0A498HA59 C Uncharacterized protein Malus domestica
NV A0A0D2MYE7 C Uncharacterized protein Gossypium raimondii
NV A0A1U8IIM7 two-pore potassium channel 5-like isoform X1 Gossypium hirsutum
NV A0A2K1ITF6 C Uncharacterized protein Physcomitrium patens
NV D8RPT8 C Uncharacterized protein SmTPK1 1 Selaginella moellendorffii
NV C4J1B8 C Two-pore potassium channel 1 Zea mays
NV A2YHF0 C Uncharacterized protein Oryza sativa subsp. indica
NV Q8LIN5 C Two pore potassium channel b Oryza sativa subsp. japonica
NV A0A287MX87 C EF-hand domain-containing protein Hordeum vulgare subsp. vulgare
NV M7YUU3 C Calcium-activated outward-rectifying potassium channel 1 Triticum urartu
NV A0A2G5CBJ3 C EF-hand domain-containing protein Aquilegia coerulea
A0A1S3YKH2 C two-pore potassium channel 1-like isoform X2 Nicotiana tabacum
NV A0A1S3Y4H6 C two pore potassium channel a-like Nicotiana tabacum
NV B8AJX4 C EF-hand domain-containing protein Oryza sativa subsp. indica
NV Q850M0 C Two pore potassium channel a Oryza sativa subsp. japonica
NV A0A317Y0N8 C Two pore potassium channel a Zea mays
NV A0A453LTY0 C EF-hand domain-containing protein Aegilops tauschii subsp. strangulata
NV M7Z2H2 C Calcium-activated outward-rectifying potassium channel 1 Triticum urartu
NV A0A287SDA1 C EF-hand domain-containing protein Hordeum vulgare subsp. vulgare
NV B8PR76 C Outward-rectifying potassium channel Hordeum vulgare subsp. vulgare
A0A1S3Y5N9 C two-pore potassium channel 1-like Nicotiana tabacum
NV A0A443N2W2 C Two-pore potassium channel 1-like protein Cinnamomum micranthum f. kanehirae
NV A0A2K2C9H1 C Ion trans 2 domain-containing protein Populus trichocarpa
NV Q8LBL1 C Two-pore potassium channel 1 Arabidopsis thaliana
NV I1MQY9 C EF-hand domain-containing protein Glycine max
NV A0A0A0K3G0 C CUCSA Uncharacterized protein Cucumis sativus
NV A0A498JVR9 C EF-hand domain-containing protein Malus domestica
NV A0A2K1X8U5 C Uncharacterized protein Populus trichocarpa
NV A0A0A0LW31 C Uncharacterized protein Cucumis sativus
